# YAP/Smad3 promotes pathological extracellular matrix microenviroment‐induced bladder smooth muscle proliferation in bladder fibrosis progression

**DOI:** 10.1002/mco2.169

**Published:** 2022-09-15

**Authors:** Xing‐Peng Di, Xi Jin, Jian‐Zhong Ai, Li‐Yuan Xiang, Xiao‐Shuai Gao, Kai‐Wen Xiao, Hong Li, De‐Yi Luo, Kun‐Jie Wang

**Affiliations:** ^1^ Department of Urology, Institute of Urology (Laboratory of Reconstructive Urology) West China Hospital, Sichuan University Chengdu Sichuan China

**Keywords:** bladder fibrosis, extracellular matrix stiffness, proliferation, Smad3, YAP

## Abstract

Fibrosis is a chronic inflammation process with excess extracellular matrix (ECM) deposition that cannot be reversed. Patients suffer from bladder dysfunction caused by bladder fibrosis. Moreover, the interactive mechanisms between ECM and bladder fibrosis are still obscure. Hence, we assessed the pivotal effect of Yes‐associated protein (YAP) on the proliferation of bladder smooth muscle in fibrosis process. We identified that stiff ECM increased the expression and translocation of YAP in the nucleus of human bladder smooth muscle cell (hBdSMC). Sequencings and proteomics revealed that YAP bound to Smad3 and promoted the proliferation of hBdSMC via MAPK/ERK signaling pathway in stiff ECM. Moreover, CUT and TAG sequencing and dual‐luciferase assays demonstrated that Smad3 inhibited the transcription of JUN. The YAP inhibitor CA3 was used in a partial bladder outlet obstruction (pBOO) rat model. The results showed that CA3 attenuated bladder smooth muscle proliferation. Collectively, YAP binding with Smad3 in the nucleus inhibited the transcription of JUN, and promoted the proliferation of bladder smooth muscle through the MAPK/ERK signaling pathway. The current study identified a novel mechanism of mechanical force induced bladder fibrosis that provided insights in YAP‐associated organ fibrosis.

## Graphical abstract

Our study revealed the effect of YAP/Smad3 in response to extracellular matrix (ECM) stiffness, which can delay or even reverse bladder fibrosis. The ECM‐induced stiffness regulating the proliferation of bladder smooth muscle cells by YAP/Smad3 will provide the basis for further in‐depth research.

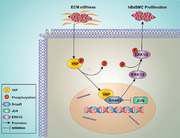

## 1 INTRODUCTION

Fibrosis is a tissue injury and repair process that highly correlated with chronic inflammation and others. In urological diseases, bladder dysfunction is associated with fibrosis. There are over 50% men over 60 years old suffer from benign prostatic hyperplasia (BPH), and 7% of these patients had urinary retention.^1^ Slight tissue injury promotes reconstruction. However, repeated severe injury can lead to tissue or organ failure.^2^ Currently, the main therapies for BPH are drug application or surgery. However, as long‐term injury to the bladder occurs, the bladder fibrosis cannot be easily cured, and the bladder function cannot recover either. Suffering from pathological mechanical force results in bladder inflammation and excess extracellular matrix (ECM) deposition, which leads to bladder fibrosis. Ultimately, upper urinary tract obstruction or hydronephrosis progress into kidney failure.^3^ And excess ECM deposition causes stiffness changes in tissue that lead to irreversible injury.

In the process of fibrosis, ECM microenvironment is altered by element change, which is called ECM stiffness.^4^ ECM stiffness is significant in promoting embryonic development, tissue repair, and cell adhesion. However, excess pathological mechanical force put fibrosis and tumors in great progression. Recent studies have demonstrated that ECM stiffness is correlated with fibrosis in several organs such as the heart, lung, and kidney.^5^, ^6^, ^7^ Severe bladder outlet obstruction (BOO) leads to irreversible damage to the bladder function.^8^ The mechanisms of BOO‐related bladder reconstruction are complicated. For this reason, bladder fibrosis is a troublesome problem that has not been solved to date.

Yes‐associated protein (YAP) can sense ECM stiffness, cell density, basement adhesion force, and 3D matrix signals to promote the disease development.^9^ YAP is also an important downstream molecule of the Hippo signaling pathway that regulates organ reconstruction, tumor progression, etc.^9^, ^10^ The currently known biomechanical force‐promoting YAP effect is manifested mainly in the vascular smooth muscle cell proliferation,^11^ epithelial damage repair,^12^ fibrosis,^13^ and others.

Smad family proteins are central transductors of the TGFβ signaling pathway. TGFβ/Smad regulates tissue and organ fibrosis mainly by activating fibroblasts.^14^ There are three types of Smad proteins: receptor regulated Smads (R‐Smads, Smad1/2/3/5/9), inhibitive Smads (I‐Smads, Smad6/7), and common mediator Smads (co‐Smad, Smad4).^15^ Moreover, the Smad related signaling pathway plays an important role in immune system diseases, malignant tumor, and neurological diseases.^16^, ^17^, ^18^

Although recent studies revealed that ECM stiffness is involved in fibrosis and malignant tumor,^19^ the exact mechanism in bladder fibrosis is still unclear. Moreover, whether ECM stiffness regulates bladder fibrosis through YAP still needs further research. In this study, YAP was regarded as a crucial transductor between ECM stiffness and smooth muscle proliferation in fibrosis progression. We aimed to identify the mechanism of ECM‐induced bladder smooth muscle proliferation that provided a novel research direction of fibrosis.

## 2 RESULTS

### 2.1 ECM stiffness‐induced human bladder smooth muscle cell proliferation is regulated by the nuclear localization of YAP

**FIGURE 1:**
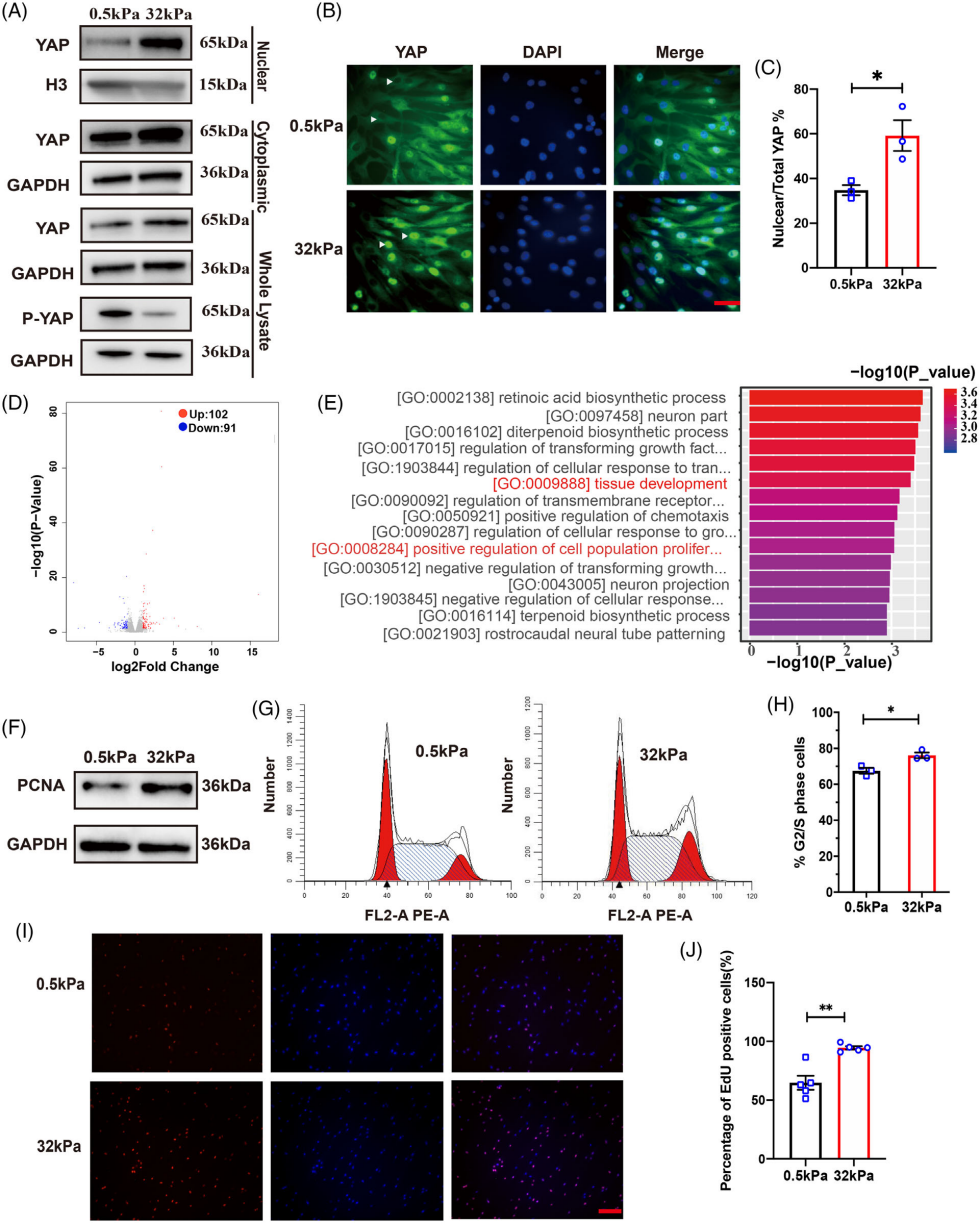
“Stiff” ECM induced YAP translocation and promoted hBdSMC proliferation. (A) Nuclear, cytoplasmic, whole YAP protein and phosphorylated YAP at Ser127 (P‐YAP^S127^) were detected by Western blot after 48 h. (B) Fluorescence imaging of YAP under different ECM stiffness was performed to identify YAP location in cells. The white arrows mean the nucleus, scale bar = 50 µm. (C) Quantification of nuclear/total YAP cell percentage in (B) is mean ± SEM, *n* = 3. *P < 0.05. (D) Total RNA transcriptome sequencing of cells under different ECM stiffness. (E) Gene ontology (GO) enrichment analysis. (F) Proliferating cell nuclear antigen (PCNA) protein of cells under different stiffness was detected by western blot. (G) Flow cytometry of cells under different stiffness. (H) Quantification of (G) is mean ± SEM, *n* = 3. *P < 0.05. (I) EdU assay of cells under different stiffness, scale bar = 200 µm. (J) Quantification of the EdU assay in (I) is the mean ± SEM, *n* = 5. **P < 0.01

A two‐dimensional silicone gel was utilized to construct a model for ECM stiffness. The cells cultured on gel can sense the force of ECM. According to previous studies,^20^, ^21^, ^22^ 0.5 kPa and 32 kPa were chosen for human bladder smooth muscle cell (hBdSMC) culture. On 32 kPa silicone gel, the protein expression of collagen I (Col I, Figure S1A) and fibronectin (Fn, Figure S1B) increased significantly compared with 0.5 kPa. PCR results showed similar results (Figure S1C,D). Moreover, the supernatants of 0.5 kPa and 32 kPa were collected for the enzyme‐linked immunosorbent assay (ELISA) test to validate the model quality in vitro. There were more Col I and Fn proteins in the ECM at 32 kPa (Figure S1E,F). As YAP is always activated by dephosphorylation and translocated into the nucleus,^23^ western blot (WB) was used to detect the P‐YAP expression in the cytoplasm. The results revealed that P‐YAP decreased significantly accompanied by total YAP increase (Figure 1A). After 24 h, the cells were fixed on a gel for immunofluorescence of the YAP test. Fluorescent imaging demonstrated a preferential nuclear accumulation on “stiff” (32 kPa) silicone gel (Figure 1B,C).

Although studies have found that ECM stiffness could promote tissue proliferation and reconstruction,^24^ the exact phenotypes regulated by ECM stiffness in bladder tissue were unknown. Therefore, a transcriptome sequencing was performed to predict the phenotype tightly related to mechanical cues. We set |log2FC| < 1 and *P*_value < 0.05 for bioinformatical analyses. There were 193 differentially expressed genes (DEGs) sheltered, among which there were 102 upregulated genes and 91 downregulated genes (Figure 1D). Gene ontology (GO) analysis was used to analyze the DEGs. The upregulated DEGs were enriched in tissue development, growth factors, positive regulation, etc. (Figure 1E). Hence, proliferation was selected as a key phenotype for further research. Moreover, the proliferation‐associated protein PCNA detected by WB on the 32 kPa gel plate also demonstrated higher expression than the 0.5 kPa gel plate (Figure 1F). The flow cytometry results revealed a significant increase in G2/S phase cells on the 32 kPa gel plate compared with the 0.5 kPa gel plate (Figure 1G,H). The 5‐ethynyl‐2′deoxyuridine staining (EdU) assay confirmed the results above as well (Figure 1I,J).

**FIGURE 2:**
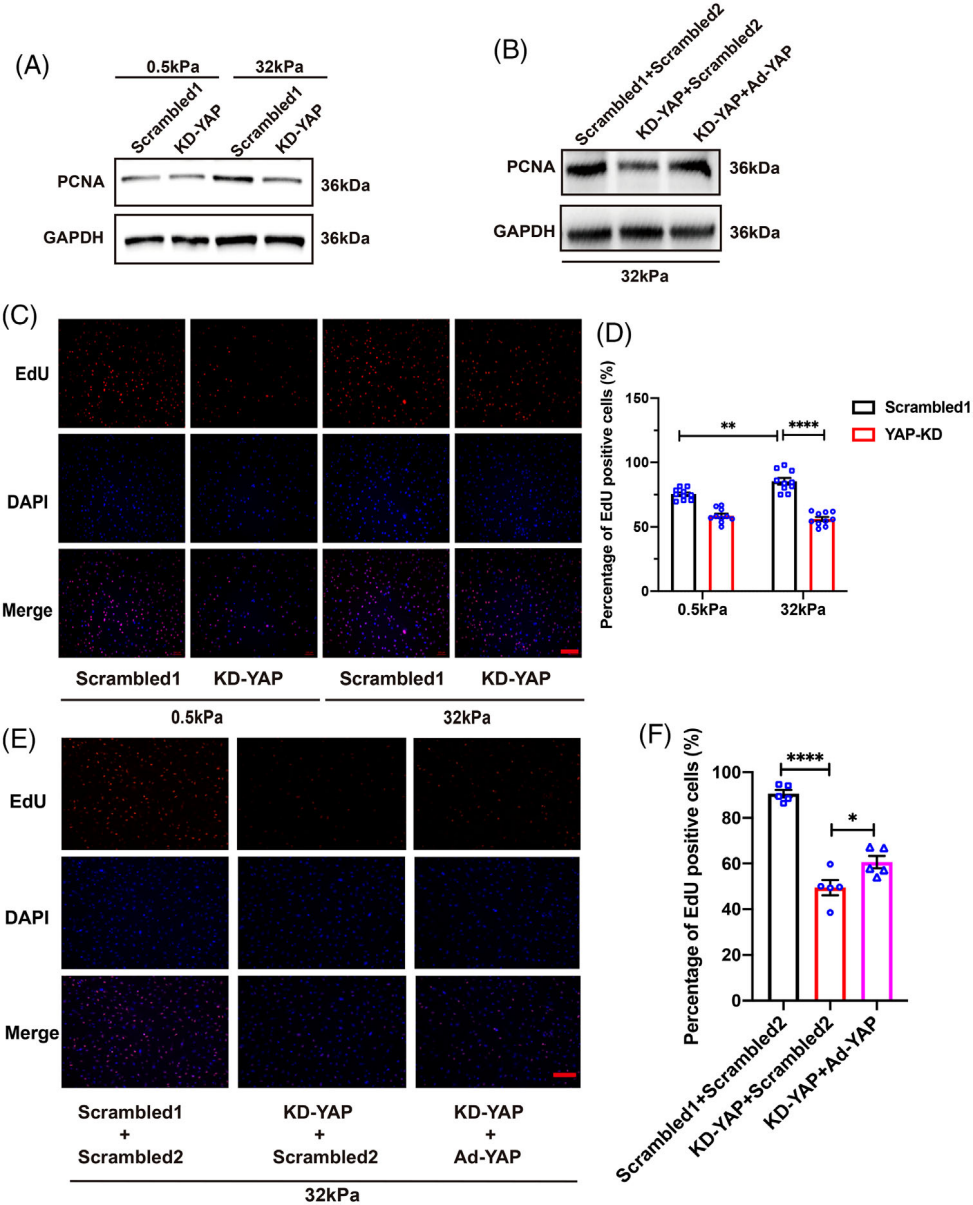
YAP is the key regulator in ECM stiffness‐induced hBdSMCs proliferation. (A) Proliferating cell nuclear antigen (PCNA) protein of cells with YAP knocked down under different stiffness was detected by western blot, *n* = 3. KD‐YAP, Knock down of YAP. Scrambled1 is for KD‐YAP. (B) PCNA was detected by Western blot under 32 kPa in rescue experiment, *n* = 3. Ad‐YAP, over‐expression of YAP. Scrambled1 is for KD‐YAP, Scrambled2 is for Ad‐YAP. (C) EdU assay of KD‐YAP under different stiffness, scale bar = 200 µm. (D) Quantification of EdU assay in (C) is the mean ± SEM, *n* = 10, **P < 0.01, ****P < 0.0001. (E) EdU assay of rescue experiment under 32 kPa was performed to detect the DNA replication, scale bar = 200 µm. Scrambled1 is for KD‐YAP, Scrambled2 is for Ad‐YAP. (F) Quantification of the EdU assay in (E) is the mean ± SEM, *n* = 5, *P < 0.05, ****P < 0.0001

The transcription coactivator YAP plays a vital role in tissue and organ development and regeneration. Several studies have identified YAP as a transductor of ECM stiffness signals.^25^, ^26^ Hence, we conducted the current research to investigate whether YAP can sense the bladder ECM stiffness changes. hBdSMCs were cultured on “soft” (0.5 kPa) and “stiff” (32 kPa) gels for 24 h. hBdSMCs were infected with adeno‐associated virus (AAV) containing YAP shRNA and overexpression vector with GFP green fluorescence. The multiplicity of infection (MOI) = 100 was validated for use through fluorescence imaging and WB (Figure S2). The results showed that PCNA protein increased at 32 kPa. When YAP was knocked down, PCNA decreased along with the change in YAP (Figure 2A). The 5‐ethynyl‐2'deoxyuridine staining (EdU) assay conducted 24 h after AAV infection provided the similar results (Figure 2C,D). Furthermore, we conducted a rescue experiment of YAP. After YAP protein was rescued by AAV infection, the PCNA protein increased significantly again at 32 kPa (Figure 2B). The EdU assay of the rescue experiment also confirmed the outcomes (Figure 2E,F).

### 2.2 ECM‐induced hBdSMC proliferation regulated by YAP is independent of the Hippo signaling pathway

As YAP always acts as an important member of the Hippo signaling pathway, the Hippo signaling pathway was selected for further research. P‐MST/MST and P‐LATS/LATS expressions were evaluated by WB. There was no difference in the P‐MST and P‐LATS expression ratios between 0.5 kPa and 32 kPa (Figure S3A–C). Furthermore, we knocked down LATS by plasmid transfection. The EdU assay revealed that DNA replication decreased due to LATS knockdown. However, MST and LATS expression did not change on “soft” or “stiff” gels. And LATS knocked down still weakened the DNA replication of hBdSMC (Figure S3D,E), partly because the Hippo signaling pathway is involved in the proliferation of hBdSMCs but independent of mechanical cues. That was consistent with previous studies.^27^, ^28^

### 2.3 YAP regulates hBdSMC proliferation through the MAPK/ERK signaling pathway

**FIGURE 3:**
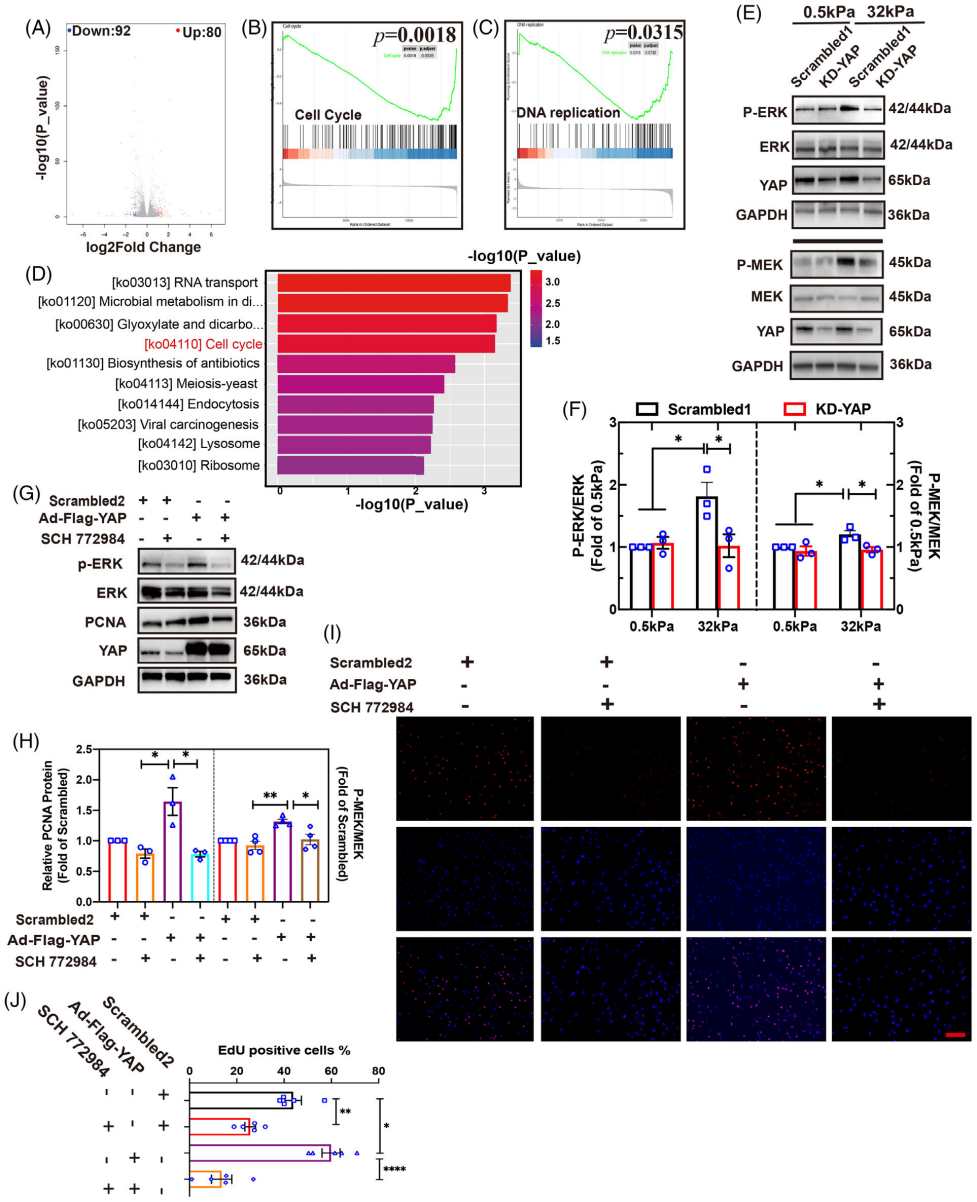
YAP regulates hBdSMC proliferation through the MAPK/ERK signaling pathway. (A) Differentially expressed genes (DEGs) of RNA transcriptome sequencing were identified after YAP was knocked down. (B,C) Kyoto Encyclopedia of Genes and Genomes (KEGG) analysis of cell cycle and DNA replication. (D) KEGG enrichment analysis for downregulated DEGs. (E) P‐ERK/ERK and P‐MEK/MEK were detected by western blot after YAP was knocked down. Scrambled1 is for KD‐YAP. (F) Quantifications of (E) are the mean ± SEM, *n* = 3, *P < 0.05. (G) YAP was over expressed, and SCH772984 was utilized. The expression of P‐ERK/ERK and proliferating cell nuclear antigen (PCNA) under 32 kPa were detected by Western blot. Scrambled2 is for Ad‐Flag‐YAP. (H) Quantifications of expression of P‐ERK/ERK (*n* = 3) and PCNA (*n* = 4) in (G) are the mean ± SEM, *P < 0.05, **P < 0.01. (I) EdU assay under 32 kPa was performed in rescue experiment, scale bar = 200 µm. Scrambled2 is for Ad‐Flag‐YAP. (J) Quantification of (I) is the mean ± SEM, *n* = 5, *P < 0.05, **P < 0.01, ****P < 0.0001

From the results above, we found that on the “stiff” (32 kPa) gel, YAP regulated hBdSMCs independent of the Hippo signaling pathway. However, are there any other pathways responding to mechanical force on cell proliferation? Hence, YAP‐jknockdown transcriptome sequencing on a “stiff” (32 kPa) gel was performed to identify potential signaling pathways. Through bioinformac analyses, 172 DEGs were identified with |log2FC|<1 and *P*‐value < 0.05. There were 80 upregulated DEGs and 92 downregulated DEGs (Figure 3A). Moreover, Kyoto Encyclopedia of Genes and Genomes (KEGG) analyses further confirmed that the cell cycle (ko04110) and DNA replication (ko03030) related MAPK signaling pathway (ko04013) was associated with mechanical force induced cell proliferation (Figure 3B,D).

P‐ERK/ERK and P‐MEK/MEK increased significantly on the “stiff” (32 kPa) gel, as shown by WB. After YAP was knocked down, P‐ERK/ERK and P‐MEK/MEK levels decreased (Figure 3E,F). The results above initially showed the relationship between YAP and the MAPK/ERK signaling pathway. Then, the ERK phosphorylation inhibitor SCH772984 was used to inhibit the MAPK signaling pathway. CCK‐8 test was conducted to identify the half maximal inhibitory concentration of SCH772984 = 10.59 µg/ml (Figure S4A). An ideal working concentration of 5 µM was confirmed by WB (Figure S4B). A rescue of YAP overexpression plus MAPK signaling pathway inhibition was conducted to ensure the relationship between YAP and MAPK under “stiff” conditions. The results demonstrated a PCNA protein increase when YAP was overexpressed. When the SCH772984 was applied, the PCNA protein level declined significantly (Figure 3G,H). The EdU assay offered a similar outcome (Figure 3I,J). Through the experiment above, we found that YAP regulated hBdSMC proliferation through the MAPK/ERK signaling pathway under “stiff” (32 kPa) mechanical conditions.

### 2.4 Identification of YAP/Smad3 interaction under different ECM stiffnesses

**FIGURE 4:**
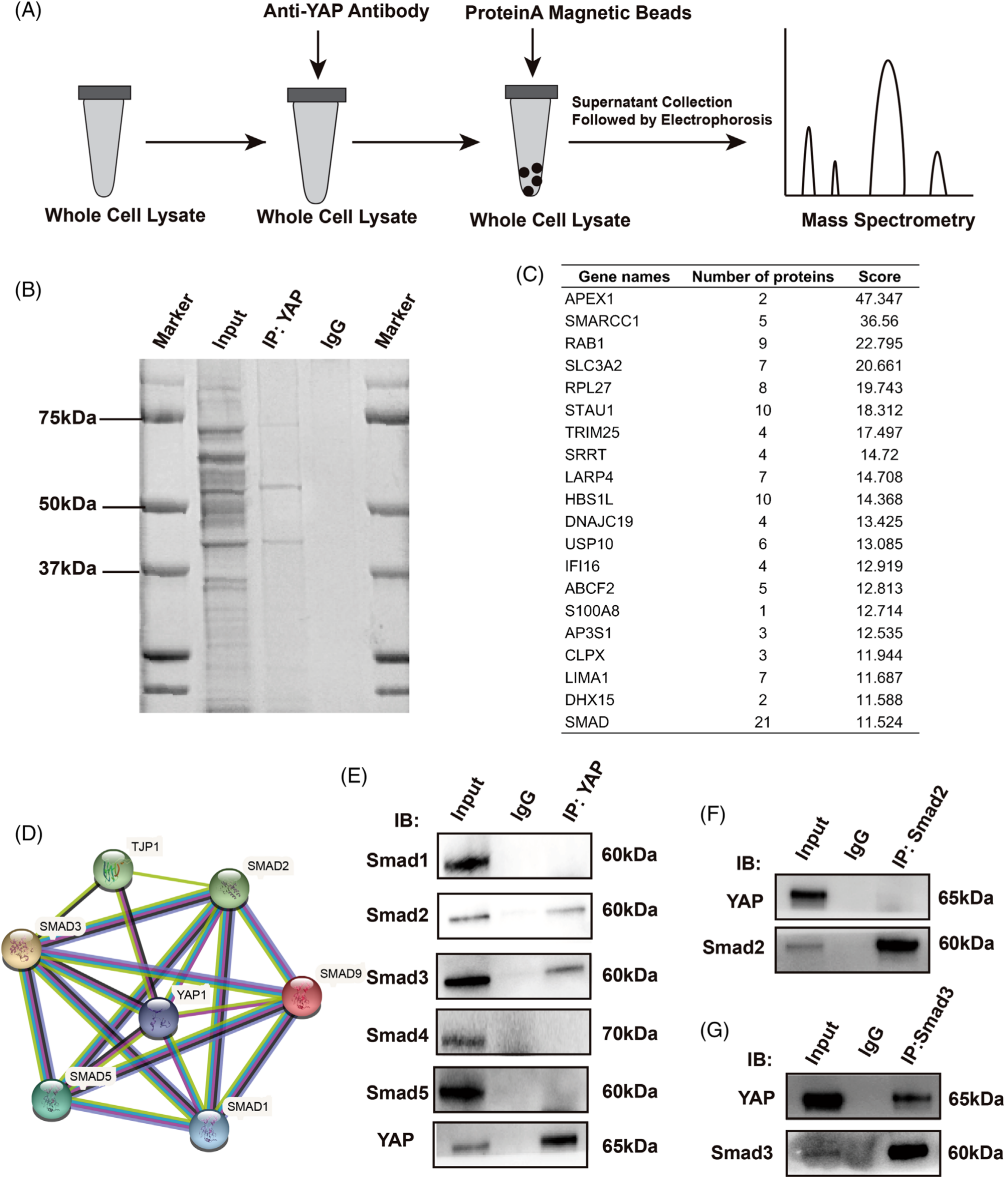
Identification of YAP/Smad3 interaction on “stiff” (32 kPa) gel. (A) A flow diagram of mass spectrometry analysis. (B) YAP immunoprecipitation was performed for electrophoresis. (C) The top 20 matching scores of peptides are listed by mass spectrometry. (D) The protein–protein interaction analyses identified the interaction between YAP and the top 20 proteins. (E) IP of YAP was performed to detect Smad1/2/3/4/5 protein by Western blot. (F) Smad2 immunoprecipitation was performed to detect YAP protein by western blot. (G) Smad3 immunoprecipitation was performed to detect YAP protein by western blot

As a transcription coactivator, YAP binds to many transcription factors. Therefore, co‐immunoprecipitation (co‐IP) of YAP and mass spectrometry experiments were performed to identify transcription factors that interacted with YAP (Figure 4A). Co‐IP experiment of whole cell lysates was conducted, and the gels were collected for mass spectrometry (Figure 4B). Then, we matched the top 20 proteins according to the peptides predicted by mass spectrometry (Figure 4C). Protein–protein interaction (PPI) analyses by STRING (https://cn.string‐db.org) were used to identify the proteins that are both transcription factors and tightly associated with YAP. Ultimately, the PPI results revealed that Smad family proteins ranked first with the highest possibility of binding to YAP (Figure 4D). The results above were further confirmed by co‐IP. IP‐YAP identified Smad2 and Smad3 as candidates (Figure 4E). A reversible Smad2 and Smad3 co‐IP test identified Smad3 as the main transcription factor binding with YAP, which provided a deeper understanding of YAP mediated proliferation under stiff mechanical cues (Figure 4F,G).

### 2.5 Smad3 binds to JUN to intervene in hBdSMC proliferation

**FIGURE 5:**
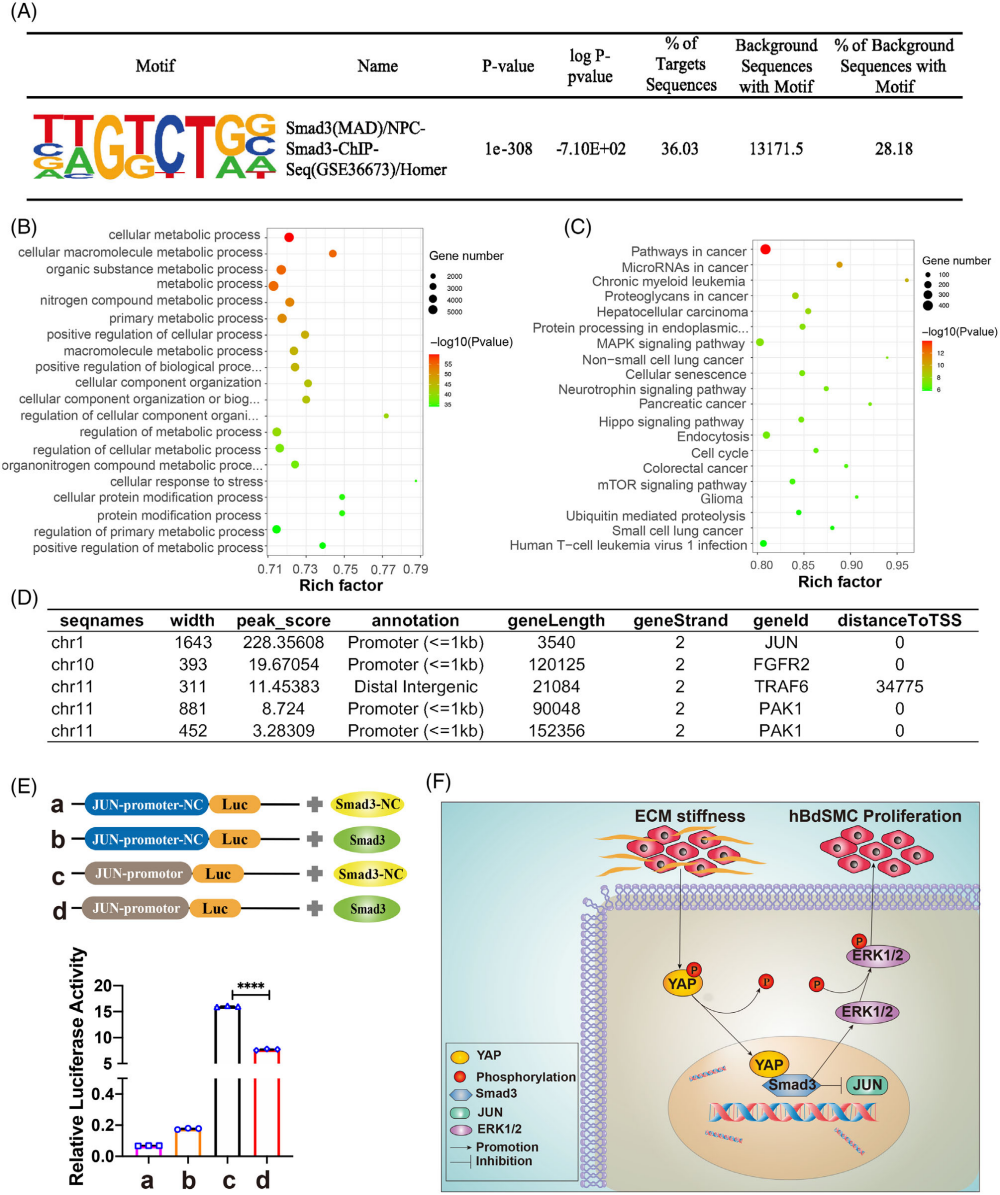
Smad3 binds to JUN to intervene in hBdSMC proliferation. (A) CUT and TAG sequencing was conducted. Smad3 was identified by specific motif from JASPAR. Gene ontology (GO) analyses were used to analyze the predicted target genes. (B) showed the biological process, analysis. (C) Kyoto Encyclopedia of Genes and Genomes (KEGG) analysis of target genes. (D) The four genes ranked by Peak_score from JASPAR. (E) The four groups in the dual‐luciferase reporter assay. NC, Natural control; Luc, Luciferase. Quantification is the mean ± SEM, *n* = 3. ****P < 0.0001. (F) Schematic overview of the effect of YAP on excess extracellular matrix ECM stiffness‐induced hBdSMC proliferation.

Based on our results, a CUT and TAG sequencing of the transcription factor Smad3 was performed to predict interactive target genes.^29^ We successfully matched Smad3 successfully through specific transcription factor‐binding site (Motif) analyses by JASPAR (https://jaspar.genereg.net, Figure 5A). GO‐enrichment analyses were performed, and the target genes were enriched mainly in the cell metabolism process, positive regulation of biological process, cytoskeleton, and protein binding (Figure 5B, Figure S5A,B). KEGG analyses revealed genes enriched in tumor, MAPK signaling pathways, etc. (Figure 5C).

The top 50 target genes associated with cell proliferation were further analyzed by PPI analysis (Figure S5C). Genes related to both the MAPK signaling pathway and smooth muscle proliferation were sheltered. Through PPI analysis, JUN, FGFR2, TRAF6, and PAK1 were identified (Figure 5D). According to the Peak_score and Smad3 marching score by JASPAR, JUN was finally selected as the most likely to bind to Smad3. In addition, a dual‐luciferase reporter assay was conducted to identify the relationship between Smad3 and JUN. Four groups were designed, and the relative luciferase activity increased significantly in JUN‐promoter‐luc plus Smad3‐natural control (NC). When Smad3 was overexpressed, the relative luciferase activity declined, which indicated that Smad3 inhibited the transcriptional initiation of JUN (Figure 5E). The above results are consistent with the current research results. In all, YAP/Smad3 played a pivotal role on ECM stiffness‐induced hBdSMC proliferation through the MAPK/ERK signaling pathway (Figure 5F).

### 2.6 YAP can regulate bladder smooth muscle in fibrosis bladder rat models

**FIGURE 6:**
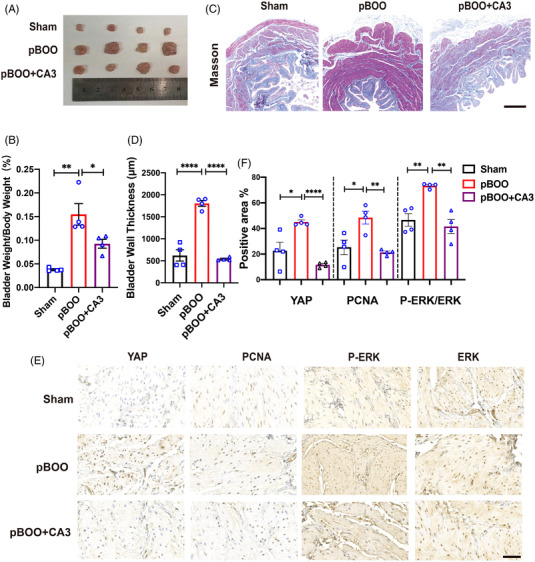
YAP promoted bladder smooth muscle in fibrosis bladder rat models. (A) Bladders of the sham operation group, partial bladder outlet obstruction (pBOO) group and pBOO+CA3 group were removed from the rats. CA3, YAP specific inhibitor, *n* = 4. (B) Bladder weight/body weight, *n* = 4, %. (C) Masson staining was used to assess the bladder muscle layer thickness, scale bar = 500 µm. (D) Quantification of bladder muscle layer thickness in (C) is the mean ± SEM, *n* = 4. ****P < 0.0001. (E) Immunohistochemistry staining of PCNA, YAP, P‐ERK, and P‐ERK were performed, scale bar = 50 µm. (F) Quantification of (E) are the mean ± SEM, *n* = 4, *P < 0.05, **P < 0.01, ****P < 0.0001

To consolidate our results in vitro, we confirmed the results in animal models. A pBOO rat model for our research^30^ and the YAP inhibitor CA3 was used in vivo. Totally, 12 rats were randomly divided into three groups (sham operation, pBOO operation, pBOO operation+CA3) and cultivated for 3 weeks under standard feeding condition. For the pBOO model construction, the medial lower abdomen was incised and a catheter with 1.2 mm diameter was introduced into the bladder. Then, the bladder neck was ligated. Finally, we removed the catheter and closed the abdomen. After 3 weeks, the bladder weight/body weight percentage was assessed. The bladder weight increased significantly in the pBOO group, while it declined when the YAP‐specific inhibitor CA3 was applied (Figure 6A,B). Besides, Masson staining was used, and we found that the bladder muscle layers thickened and more ECM was deposited, which were reversed by the application of CA3 (Figure 6C,D). Furthermore, immunohistochemistry (IHC) was performed to detect YAP, PCNA, and P‐ERK/ERK expression. The results showed that the positive area of these proteins increased in the pBOO group and decreased significantly when CA3 was applied (Figure 6E,F). Hence, the in vivo experiment initially confirmed the relationship between YAP and mechanical force induced bladder fibrosis.

In conclusion, we found that activated YAP promoted ECM stiffness‐ induced bladder smooth muscle proliferation through the MAPK/ERK signaling pathway, which was independent of the Hippo signaling pathway. In addition, JUN might be the target gene regulated by YAP in inhibiting hBdSMC proliferation. Additionally, the YAP specific inhibitor CA3 alleviated bladder muscle proliferation in a bladder fibrosis rat model.

## 3 DISCUSSION

Bladder fibrosis caused by lower urinary tract obstruction affects the quality of life of patients. Pathological mechanical force is one of the main factors of bladder fibrosis. Our previous research revealed that both periodic pathological hydrostatic pressure and stretching force induce bladder fibrosis.^31^, ^32^

In the current study, we identified the role of ECM stiffness in bladder fibrosis. And this study discovered and confirmed that pathological ECM stiffness translocated YAP into the nucleus, more nuclear YAP interacted with P‐Smad3 to activate the MAPK/ERK signaling pathway. In addition, Smad3 inhibits the transcription of JUN and ultimately promotes the proliferation of hBdSMC. Furthermore, the rat pBOO model confirmed that YAP can regulate the bladder smooth muscle thickening. Therefore, YAP might be a potential target in preventing fibrosis progression.

The ECM is in dynamic changes all the time, which is especially critical in cell development, differentiation, and death. Pathological ECM stiffness can accelerate the progression and metastasis of malignant tumours.^19^ However, there remains a lack of research on ECM stiffness in the urinary system. To solve this problem, our study explained how cells respond to mechanical cues such as ECM stiffness, and how the mechanical response affects cell behavior, providing a new direction for the research field of bladder fibrosis.

At present, whether the Hippo signaling pathway can respond to mechanical signals is controversial.^33^ The Hippo signaling was first discovered in Drosophila and mainly plays a role in the embryonic development and organ regeneration.^34^ YAP acts as a main downstream molecule of Hippo. A study revealed that the Ras‐related ATPase RAP2 can regulate cell growth by inhibiting Hippo‐YAP/TAZ in response to ECM stiffness.^27^ However, can ECM stiffness truly regulate the molecular biological behavior of YAP through Hippo? Our results are consistent with some studies that YAP is not only regulated by the Hippo signaling pathway, but also independent of mechanical cues. In contrast, translocation of YAP without intercellular junctions has been shown not to be affected by the F‐actin‐myosin‐phosphorylated YAP pathway.^35^ The current experiment revealed that in the bladder fibrosis model, mechanical force cannot induce the Hippo kinase cascade. Several studies have discussed the relationship between YAP and Smad family. Previous studies have revealed that Hippo/YAP can interact with TGFβ/Smad3 to promote tissue fibrosis.^36^ We also identified that ECM stiffness can induce YAP translocation and directly interact with Smad3 to promote transcription.

Smad family proteins have two MH domains with less conserved linkages between them. The N‐terminal MH1 domain includes the hairpin structure Smad binding element responsible for DNA binding. In addition to transcription factors, other proteins can also bind to or even modify Smad proteins. Smad proteins can also promote tissue and organ proliferation through interaction. Consistent with our outcomes, YAP can combine with Smad3 to promote the proliferation of hBdSMC. In this study, we predicted many proteins that can bind to YAP. And PPI analysis identified an interaction between Smad and YAP, which was further confirmed by co‐IP experiment. Subsequently, we confirmed that Smad3 may have a regulatory effect on the cell cycle‐related target gene JUN through the results of CUT and TAG sequencing and dual‐luciferase reporter assays.

JUN is a proto‐oncogene first discovered in the genome of avian sarcoma virus 17(ASV17).^37^ JUN is currently repeated to be involved in the embryonic brain development, cell‐cycle regulation, apoptosis, and axis formation.^38^, ^39^, ^40^ Some studies have reported that the N‐terminal kinase JUN N‐terminal kinases (JNK) of JUN can activate the MAPK signaling pathway to regulate drug sensitivity and tumor resistance.^41^ In our study, CUT and TAG sequencing and dual‐luciferase reporter assay demonstrated that Smad3 might inhibit the transcription of JUN which is contrary to the conclusions of some current studies. Studies have also shown that the expression levels of the N‐terminal kinases JNK and p‐c‐JUN of c‐JUN are significantly reduced when anti‐tumor drugs are used, which means that JUN plays a role in regulating cell‐cycle escape.^42^ Therefore, JUN may also play a similar role in our study, which is consistent with some existing studies.

Fibrosis is related to a variety of signaling pathways, such as TGFβ/SMAD signaling pathway, integrin signaling pathway, MAPK signaling pathway, ROCK Rho‐related signaling pathway, etc. Current studies have shown that Hippo/YAP can interact with proteins to regulate vascular smooth muscle proliferation.^43^ In addition, TGFβ1 promotes the proliferation of airway smooth muscle cells by promoting the expression of transient receptor potential melastatin 7 through the TGFβR/Smad3 signaling pathway.^44^ The Wnt signaling pathway regulates the embryonic development and smooth muscle cell proliferation, migration and survival through β‐catenin activation.^45^ In addition, the MAPK/ERK signaling pathway is currently known to be closely related to the cell cycle. In our study, the results of transcriptomic sequencing after YAP knockdown revealed that the MAPK/ERK signaling pathway regulated cell proliferation. As we confirmed that YAP interacts with Smad3, follow‐up studies are needed to explore the relationship between Smad3 and the pathways above.

Our study inevitably has some limitations. Regarding the selection of mechanical parameters, this study refers to the existing research on 2D hydrogels. At present, 0.5 kPa and 32k Pa are mostly used to simulate the difference in matrix stiffness. In future research, we will determine more suitable and accurate mechanical parameters. In addition, this study concluded that Smad3 might inhibit the transcription of JUN and thereby inhibit the cell cycle of hBdSMC, which is controversial in some studies. Therefore, research is needed to confirm the relationship between Smad3 and c‐JUN in affecting the cell cycle. As bladder fibrosis is a benign disease, it is difficult to obtain clinical specimens, which is one of the main limitations of this study.

In conclusion, our study explored how ECM stiffness regulates the proliferation of hBdSMC through YAP/Smad3. YAP regulates ECM‐induced bladder smooth muscle proliferation through the MAPK/ERK signaling pathway. However, mechanical signals often have crosstalk between each other, so the YAP regulatory mechanism is far more than that. In addition, our study also expands the research of biomechanical force in the field of bladder fibrosis. The studies of mechanisms in organ fibrosis will support better therapies in the future.

## 4 MATERIAL AND METHODS

### 4.1 Reagents

The silicon six‐well plates of 0.5 kPa and 32 kPa were purchased from Advanced Biomatrix, USA (Cat. No: 5140‐5EA and 5144‐5EA). Smooth muscle cell medium (SMCM) was from ScienCell, USA (Ca.t No. 1101). The Quantinova SYBR Green PCR Kit was from QIAGEN, Germany (Cat. No. 208054). Hoechst 33342 was from Solabrio, China (Cat. No. C0030). The YAP inhibitor CA3 was from Selleck, China (Cat. No: S8661).

### 4.2 Preparation of pre‐coated substrate

The gel plates were incubated overnight at 4°C with 50 µg/cm^2^ of type I collagen (Advanced Biomatrix, USA). Then, all gel surface were washed with phosphate buffered saline (PBS), and the cells were seeded on top of the plates.^46^

### 4.3 Cell line and cell culture

hBdSMCs (ScienCell, USA, Cat No. 4310) were cultured with SMCM medium (ScienCell, USA, Cat. No. 1102) containing fetal bovine serum (10%), treptomycin (100 µg/ml), and penicillin (100 U/ml), and smooth muscle cell growth factor.^47^, ^48^

The 293T cells supplied by Genechem, Shanghai, were cultured with Dulbecco's modified Eagle medium (DMEM) containing fetal bovine serum (10%), treptomycin (100 µg/ml), and penicillin (100 U/ml).

All cells were incubated at 37°C and 5% CO_2_. The 3rd to 7th generations and 70%–80% cell density were the best for experiments.

### 4.4 Total RNA extraction and real‐time PCR

Total RNA was extracted using a RaPure Total RNA Kit (Magen, Guangzhou, Cat. No. R4011‐02). RNA (500 ng) was reverse‐transcribed using a RevertAid First Strand cDNA Synthesis Kit (Thermo Fisher Scientific, USA, Cat. No. 21059). Real‐time PCR was conducted using a QuantiNova SYBR Green PCR Kit (QIAGEN, Germany, Cat. No. 208054). All the data were normalized to GAPDH, and the primer sequences are shown in Table S1.

### 4.5 Extraction of cytoplasmic and nuclear proteins

Whole cell lysates were extracted with enhanced lysis buffer containing protease inhibitor and phosphorylase inhibitor (Abmole, USA, Cat. No. M7528). Cytoplasmic and nuclear proteins were extracted by MinuteTM Cytoplasmic and Nuclear Fraction Kit (Invent, USA, Cat. No. SC‐003) containing protease inhibitor and phosphorylase inhibitor.

### 4.6 Western blot

WB was performed based on a standard protocol. The primary antibodies were from Cell Signaling Technology (CST, USA), Abcam (ab, USA), and Bioss (bsm, Beijing), as shown in Table S2. The membranes were incubated at 4°C for a night shift. Then, we incubated the membranes with the secondary antibody. The horseradish peroxidase‐conjugated secondary antibody was goat antirabbit (CST, Cat. No. 7074S) and the concentration was 1:5000. Finally, proteins were visualized by UltraSignal enhanced chemiluminescence (ECL; 4A Biotech, Beijing, Cat. No. 4AW011‐100) on Bio‐rad, ChemiDoc MP, USA. The intensity of protein bands was measured by density of autoradiogram with National Institutes of Health (NIH) ImageJ (http://rsb.info.nih.gov/ij/).

### 4.7 YAP recombinant adeno‐associated virus construction and cell infection

To knock down YAP in hBdSMC, AAV with green fluorescent and short hairpin RNAs (shRNAs) targeting YAP was from GeneChem (Shanghai). hBdSMCs were infected with adenovirus and scrambled vector at a MOI of 100 for 8 h with minimal toxicity.

### 4.8 Immunofluorescence staining

Cells cultured in six‐well plates with 0.5 kPa and 32 kPa were infected with indicated adenovirus for 48 hours. Cells were washed with precooled PBS, and then fixed in 4% paraformaldehyde. After 15 min, the cells were permeabilized with 0.1% Triton X‐100 in PBS and then blocked with 1% bovine serum albumin (BSA). Moreover, the cells were incubated with YAP primary antibody and then with Alexa fluor‐conjugated secondary antibody. After the wells were washed, we stained the DNA for 1 min. Images were visualized with a Zeiss inverted fluorescence microscope (Germany, D1/AX10 cam HRC).

### 4.9 Masson staining

pBOO rat models were constructed as described above. The bladders were fixed with paraffin for slide preparation. After the slides were dehydrated, the Masson staining was guided by standard protocols.

### 4.10 Co‐immunoprecipitation

We added cell lysate to each well and then incubated them on ice for 5 min. After transferring the cells into a precooled 1.5 ml centrifuge tube, the cells were disrupted with an ultrasonic cell disrupter 15 s for three times. Next, we added 200 µl of cell lysate to 20 µl of ProteinA magnetic beads and mixed them. The tubes were placed on the magnetic bead absorption rack and supernatants were collected. The cell lysate concentration was adjusted to 1 µg/µl. Then, we added the YAP (CST, Rabbit, Cat. No. 14074S), Smad2 (CST, Rabbit, Cat. No. 5339S), and Smad3 (CST, Rabbit, Cat. No. 9523S) primary antibodies to the cell lysate individually. And an equal amount of isotype control (CST, Cat. No. 2729S) was used as a negative control. Then, we added 20 µl of ProteinA magnetic beads (CST, Cat. No. 73778S). The centrifuge tubes were placed on the magnetic bead absorption rack. And the magnetic beads were washed five times. After discarding the supernatant, sample buffer was used to precipitate the target protein. Finally, the mixture was loaded at 100°C, and we obtained the target proteins.

### 4.11 ELISA analysis

The supernatant of different treated six‐well plates was collected. Then, the prepared ELISA reagent for Col I (Human Pro‐collagen I alpha1 ELISA Kit, Abcam, USA, Cat. No. 210966) and Fn (Human Fibronectin ELISA Kit, Abcam, USA, Cat. No. 219046) was applied for the experiment according to the provided standard protocols. After the reaction was stopped, we measured the absorbance with microplate reader (BioTek, USA, OD = 450 nm). The concentration was calculated by a standard curve.

### 4.12 EdU assay

EdU assays were conducted based on the standard protocol with an EdU Detection Kit (Rib‐BIO, Guangzhou, Cat. No. R11053.8). Cells were incubated in diluted EdU (5000:1). Then, we fixed the cells with paraffin for 15 min. Next, the Apollo reagent was applied to stain the cell for 30 min. After DNA staining for 1 min, the cells were observed with a Zeiss inverted fluorescence microscope (Germany, D1/AX10 cam HRC).

### 4.13 Cell counting kit (CCK‐8)

The CCK‐8 experiment was conducted under the guidance of standard protocol (Abmole, USA, Cat. No. M4839). Cells were incubated with diluted reagent for nearly 4 h, and the absorbance was detected by microplate reader (BioTek, USA, OD = 450 nm). Finally, the cell activity was calculated.

### 4.14 Flow cytometry

After washing the cells with PBS, precooled 70% alcohol was applied to fix the cells. Then, the cells were incubated in staining reagent (RNase:PI = 1:9, KeyGene, China, Cat. No. KGA512) for 1 h. The absorbance was measured by Cytoflex (Beckman, USA) at 488 nm.

### 4.15 Mass spectrometry

YAP and IgG primary antibodies were used for co‐IP. The target proteins were subjected to gel electrophoresis. The gel was stained with Coomassie brilliant blue for 10 min and washed with distilled water. The mass spectrometry experiment was performed with the support of *Bioprofile* Company, Shanghai.

### 4.16 Luciferase reporter assay

293T cells were cultured by standard protocol. 293T cells were used for Smad3 and JUN plasmids transfection. After 48 h of transfection, we dissolved the Luciferase Assay II solution, then added it to the Luciferase Assay substrate, dissolved the substrate, and stored it at −80°C. The medium was aspirated, and 300 µl of lysis buffer was added. Then, we introduced lysate into the detection plate, added luciferase reaction solution, and immediately used a microplate reader to detect the fluorescence value of firefly luciferase. The experimental methods and techniques were supported by GeneChem, Shanghai.

### 4.17 Plasmid construction

For construction of the LATS knockdown plasmid: the shRNA sequence for knockdown of LATS1 was GCAAGTCACTCTGCTAATT, the shRNA sequence for knockdown of LATS2 was GCAGATTGTGCGGGTCATT, and the control siRNA sequence (scrambled) was TTCTCCGAACGTGTCACGT. Construction of JUN and Smad3 overexpression plasmids were provided by GeneChem, Shanghai.

### 4.18 CUT and TAG sequencing

CUT and TAG sequencing was used to predict target genes which was supported by Jiayin Biomedical Technology, Shanghai. The experiment is an improvement of Chip‐seq and can be conducted with only 10^5^ cells.^29^, ^49^ The motif matching is based on JASPAR (https://jaspar.genereg.net). GO functional‐enrichment analysis (http://www.geneontology.org) ^50^ and KEGG pathway analysis^51^ were conducted on DEGs with a Bonferroni‐corrected *P* ≤ 0.05.

### 4.19 Transcriptome sequencing and analysis

There were two transcriptome sequencing in our study. The groups were 32 kPa versus 0.5 kPa and control versus YAP knocked down. Transcriptome sequencing and bioinformatic analyses were performed with the support of Bioprofile, Shanghai. Total RNA was extracted based on the protocol above. GO enrichment analysis (http://www.geneontology.org) ^50^ and KEGG pathway analysis^51^ were conducted on DEGs with a Bonferroni‐corrected *P* ≤ 0.05.

### 4.20 Animal model construction

Six‐week healthy female Sprague–Dawley (SD) rats were purchased from Chengdu Dossy Experimental Animal Center. All the experimental animals were fed under the condition of regular 12 h day and night in a controlled environment condition with free diet and water. The animals were assigned into the sham operation group (*n* = 4), the pBOO group (*n* = 4), and the pBOO+CA3 group (*n* = 4). CA3 is an inhibitor of YAP. All rats were injected with 0.9% chloral hydrate (3 ml/kg). Next, the midline of the lower abdomen was incised. The proximal urethra was exposed after the bladder was found. Then, a urethra catheter (outer diameter = 1.2 mm) was introduced into the bladder. Then, the proximal urethra was ligated with 4‐0 silk. Finally, the incision was closed layer by layer after removing the catheter. The sham group underwent the same procedure without urethra ligation. Then, 150 mg/kg ampicillin was applied 3 days after surgery. Bladders were collected after 3 weeks.^30^

### 4.21 Statistical analysis

All experiments were repeated in at least three biological replicates. All data are presented as the mean ± standard error of mean (SEM). Graphpad Prism (version8.00; Graphpad software Inc., CA, USA) was applied. Two‐tailed, unpaired Student's *t*‐test or one‐way analysis of variance followed by Duncan multiple comparison test were utilized to analyze the difference. *P* < 0.05 was considered statistically significant.

## CONFLICT OF INTEREST

The authors declare that there is no conflict of interest that could be perceived as prejudicing the impartiality of the research reported.

## ETHICS STATEMENT

All animal experiments were carried out with the permission of Medical Ethics Committee of West China Hospital, Sichuan University, China (2021227A).

## AUTHOR CONTRIBUTIONS

Xing‐Peng Di and Xi Jin contributed equally to this work, designed and conducted experiments, analyzed data and wrote the article. Kun‐Jie Wang and De‐Yi Luo provided conceptualization, supervision and methodology. The bioinformatics analyses were performed and checked by Li‐Yuan Xiang and Jian‐Zhong Ai. Xiao‐Shuai Gao, Kai‐Wen Xiao, and Hong Li assisted with experiments and manuscript review. All authors read and approved the final manuscript.

## Supporting information



Supplementary Materials

## Data Availability

The proteomic data have been deposited to the ProteomeXchange Consortium via the iProX partner repository with the dataset identifier PXD033188. The RNA sequence data have been submitted to the Genbank database under accession number PRJNA856421 (http://www.ncbi.nlm.nih.gov/bioproject). All the data are available from the corresponding authors upon reasonable request.
