# Supplementary material for: YAP/Smad3 promotes pathological extracellular matrix microenviroment‐induced bladder smooth muscle proliferation in bladder fibrosis progression
Source: MedComm (2020). 2022 Sep 15;3(4):e169. doi: 10.1002/mco2.169 (PMC9477793; doi:10.1002/mco2.169)
Supplement: Supplementary file 1 — Supplementary Materials [file MCO2-3-e169-s001.docx]

**YAP/Smad3 Promotes Pathological Extracellular Matrix Microenviroment-induced Bladder Smooth Muscle Proliferation in Bladder Fibrosis Progression**

Xing-Peng Di^1^, Xi Jin^1^, Jian-Zhong Ai^1^, Li-Yuan Xiang^1^, Xiao-Shuai Gao^1^, Kai-Wen Xiao^1^, Hong Li^1^, De-Yi Luo^1,^*, Kun-Jie Wang^1,^*.

^#^Xing-Peng Di and Xi Jin contributed equally to this work.

**Affiliation**:

1. Department of Urology, Institute of Urology (Laboratory of Reconstructive Urology), West China Hospital, Sichuan University, Chengdu, Sichuan, People's Republic of China.

*** Corresponding authors:**

Dr. Kun-Jie Wang. Tel: 86-28-8542-2449, E-mail: [wangkj@scu.edu.cn](mailto:wangkj@scu.edu.cn);

Dr. De-Yi Luo. Tel: 86-28-8542-3446, E-mail: luodeyi1985@163.com.

**Supplementary Materials**

**
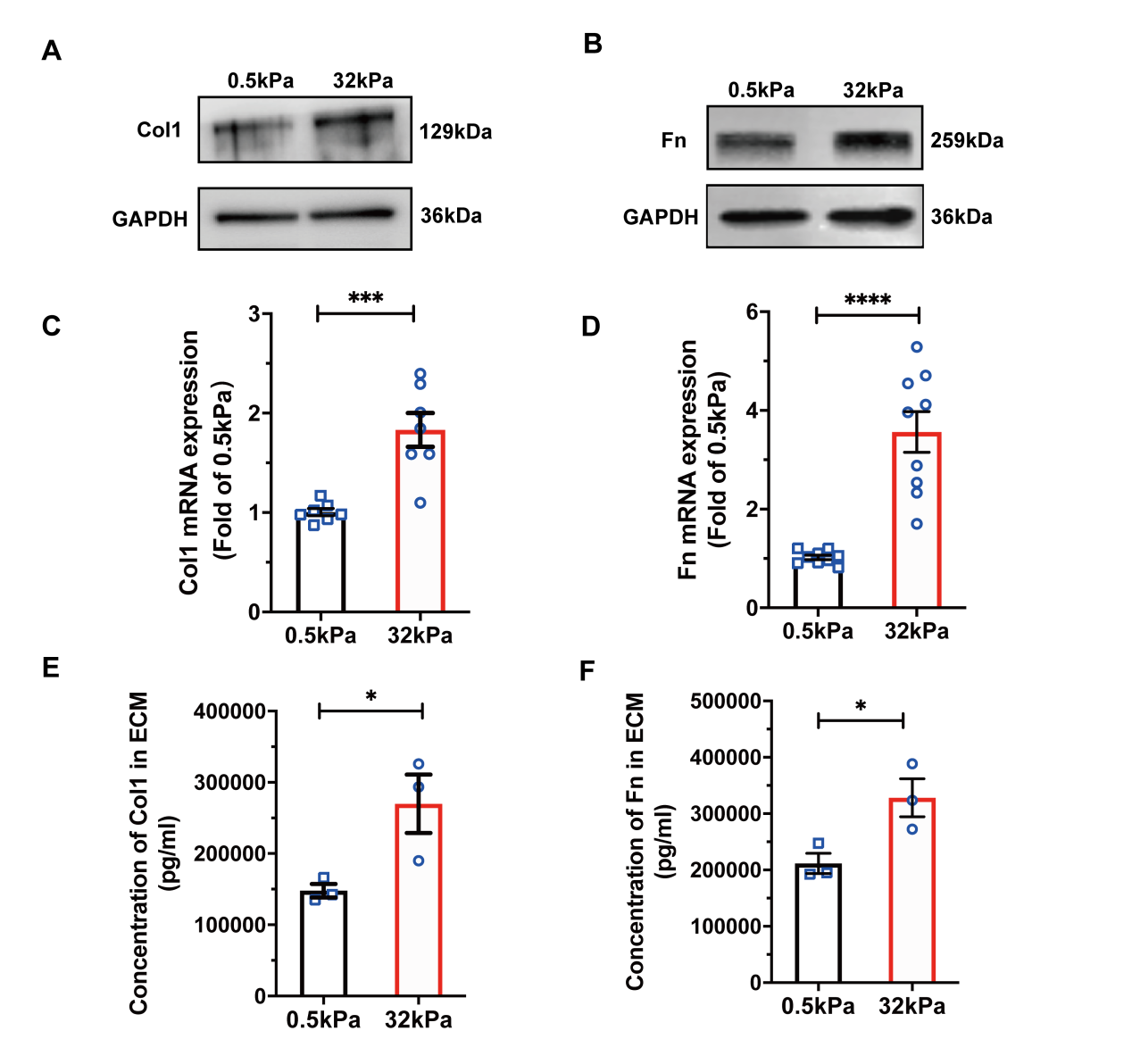
FigureS1. The in vitro models were successfully constructed on “soft” (0.5 kPa) and “stiff” (32 kPa) gels. A and B.** Collagen1 (Col I) and fibronectin (Fn) proteins were detected by Western blot under different stiffness. **C and D.** Collagen1 and fibronectin RNA were detected by PCR under different stiffness. Quantification of the expression of Col I mRNA (n=7) and Fn mRNA (n=9) are mean±SEM, ****P*<0.001, ****P*<0.0001. **E and F.** ELISA was performed to detect Col I and Fn in the extracellular matrix. Quantification of Col I and Fn are mean ± SEM, n=3, **P*<0.05.

**
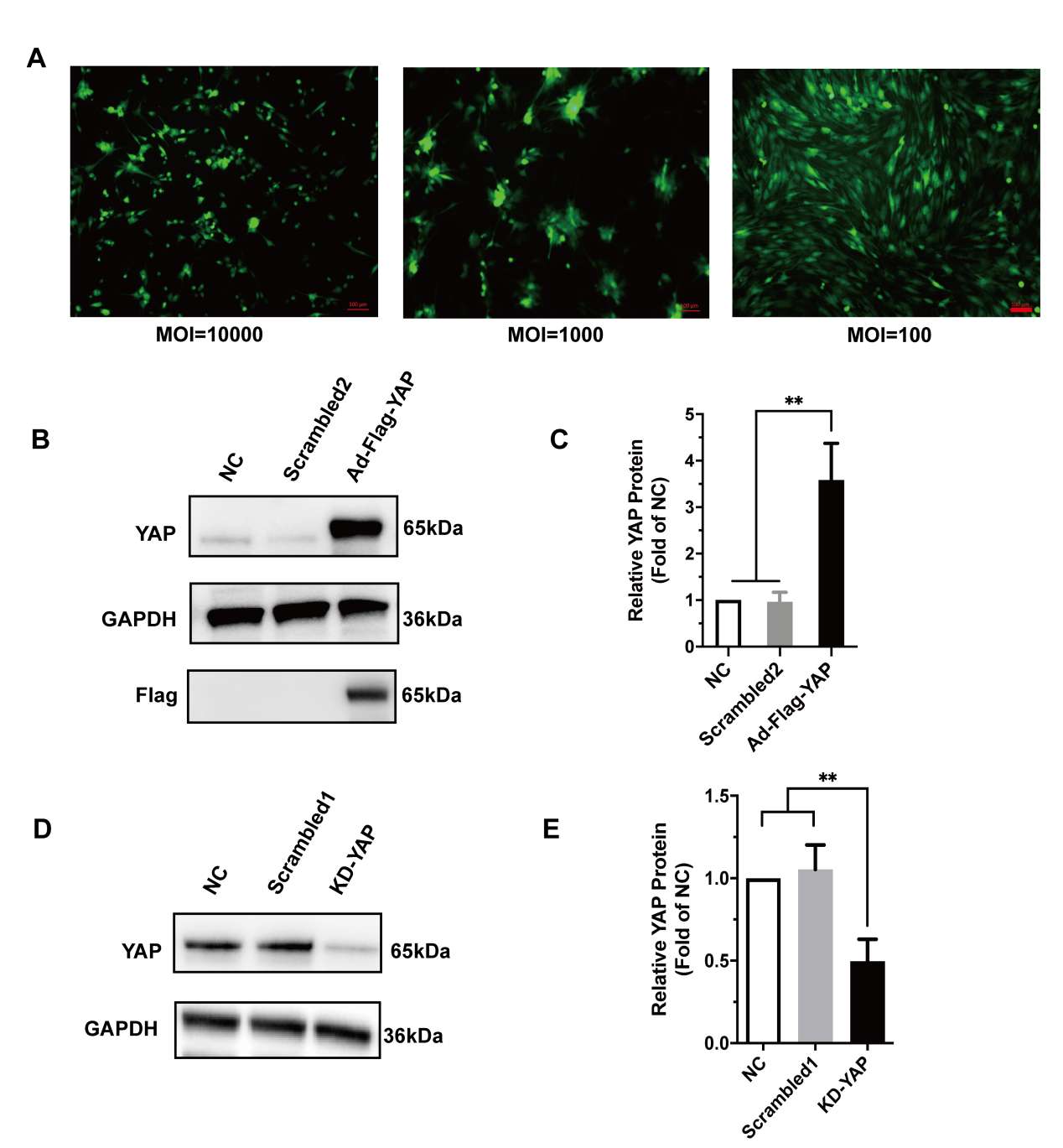
FigureS2.** **Adeno-associated virus (AAV) of YAP knockdown (KD-YAP) and overexpression (Ad-Flag-YAP) efficacy of infection. A.** Adeno-associated virus of YAP overexpression (Ad-Flag-YAP) and knockdown (KD) at multiplicity of infection (MOI)=100 were applied after 24 hours. Fluorescent imaging was used to assess the efficacy of AAV infection. Scale bar=100 μm. **B.** YAP and Flag proteins were detected by Western blot. Scrambled2 is for Ad-Flag-YAP.**C.** Quantification of YAP in **B** is mean ± SEM, n=3, NC = Natural Control, ***P*<0.01.Scrambled2 is for Ad-Flag-YAP. **D.** YAP protein was detected by Western blot after YAP was knocked down. Scrambled1 is for KD-YAP. **E.** Quantification of YAP in **D** is the mean ± SEM, n=3, NC = Natural Control, ***P*<0.01.Scrambled1 is for KD-YAP.

**
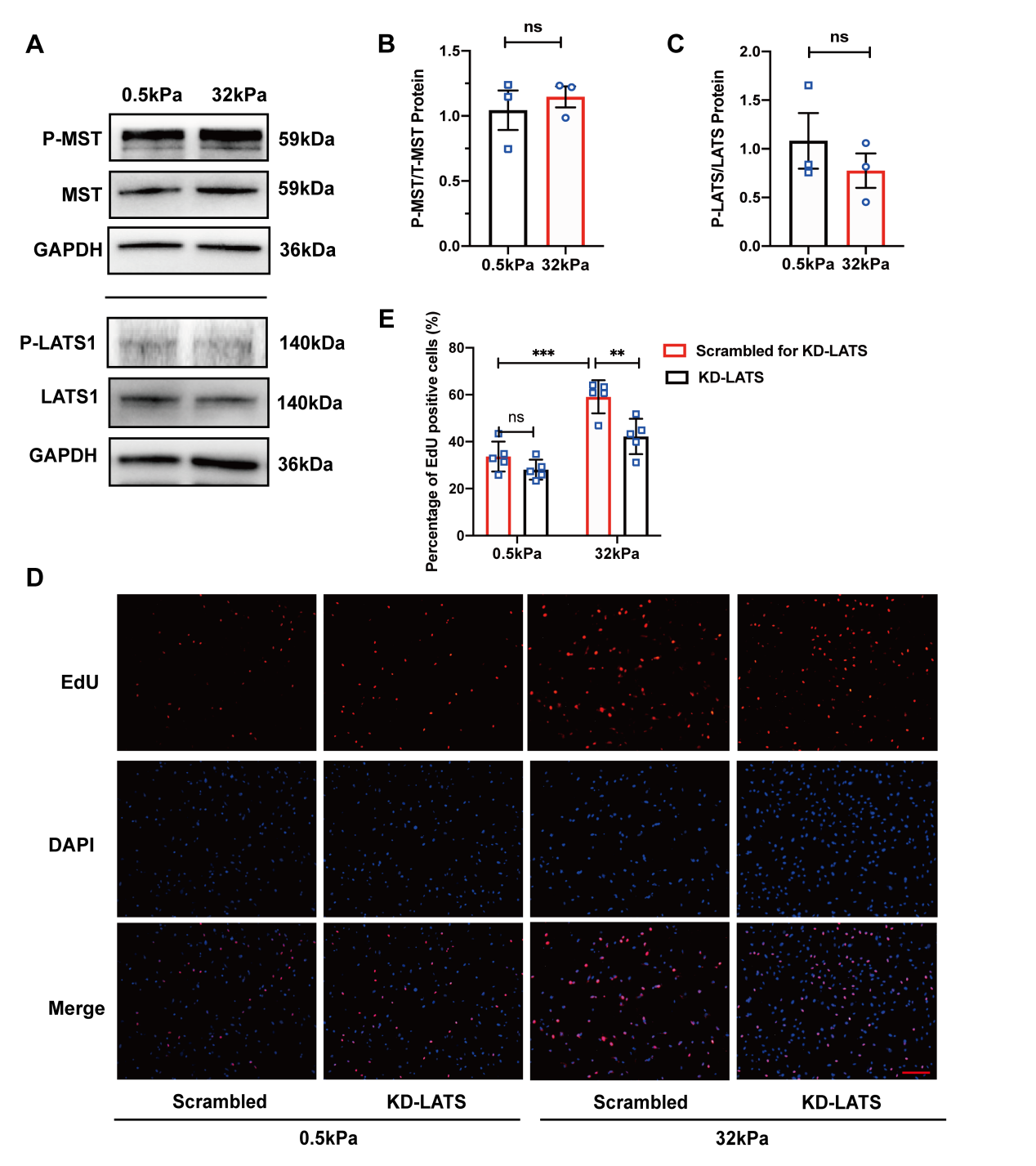
**

**FigureS3. YAP on ECM stiffness-induced hBdSMC proliferation independent of the Hippo signalling pathway. A.** The protein expression of P-MST/MST and P-LATS1/LATS1 under different stiffness were detected by Western blot. **B and C.** Quantification of the expression of P-MST/MST and P-LATS/LATS are mean ± SEM, n=3, ns=no significance. **D.** The plasmids for LATS knockdown (KD-LATS) were transfected into cells after 24 hours. EdU assay was performed to detect the DNA replication, scale bar=200 μm. **E.** Quantification of EdU assay is mean ± SEM, n=5, ***P*<0.01, ****P*<0.001.

**
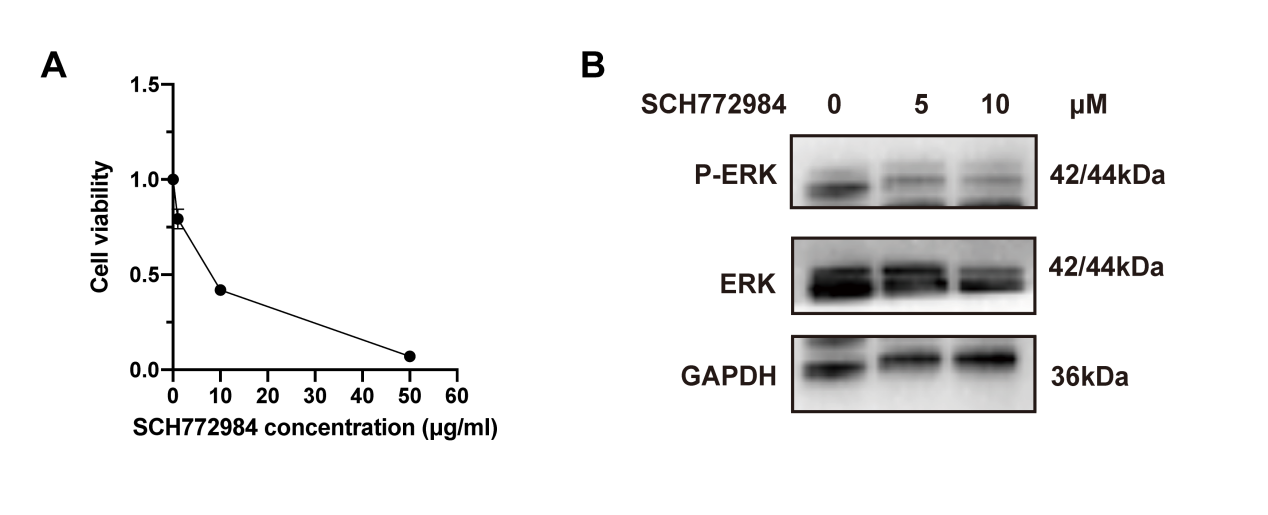
FigureS4. A.**The half maximal inhibitory concentration of SCH772984 was identified by CCK-8 test, n=5. **B.** The expression of P-ERK/ERK was detected by Western blot after SCH772984 was applied.

**
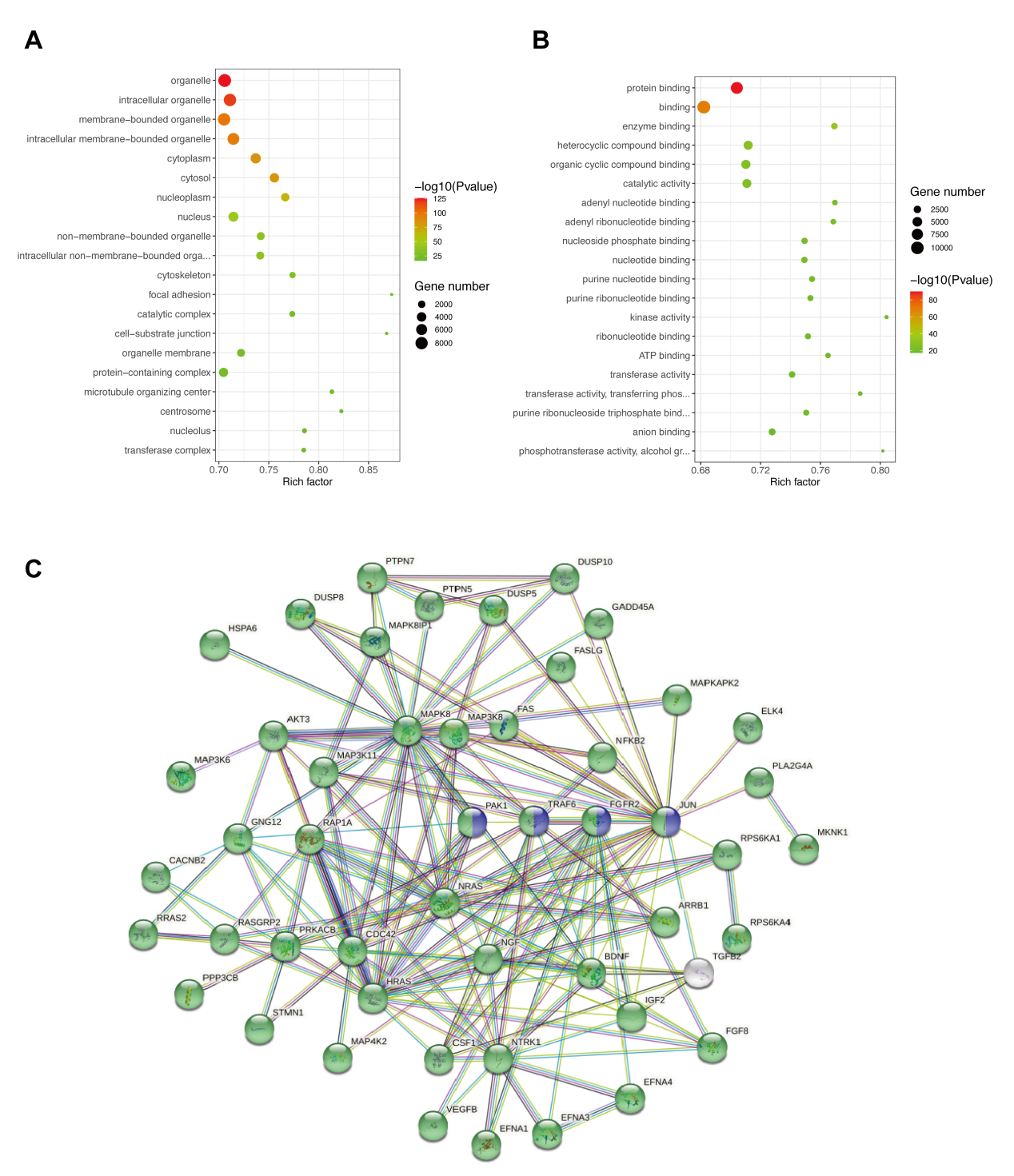
**

**FigureS5**. **A** and **B** showed the cellular component and molecular function analyses of Gene Ontology (GO) analyses. **C.** Top 50 target genes were identified by protein-protein interaction analysis. The green and purple patterns were genes both related to both the MAPK signalling and smooth muscle proliferation.

**TableS1. Primer sequences**

| **Name** | **Specimen** | **Sequence** |
| --- | --- | --- |
| Collagen I | Homo sapien | Forward: GTGCGATGACGTGATCTGTGA |
|  |  | Reverse: CGGTGGTTTCTTGGTCGGT |
| Fibronectin | Homo sapien | Forward: GGAGAATTCAAGTGTGACCCTCA |
|  |  | Reverse: TGCCACTGTTCTCCTACGTGG |
| GAPDH | Homo sapien | Forward: ACAACTTTGGTATCGTGGAAGG |
|  |  | Reverse: GCCATCACGCCACAGTTTC |

**TableS2.** **Primary and secondary antibodies for Western Blot**

| **Target** | **Concentration** | **Cat Number** |
| --- | --- | --- |
| YAP | 1:1000 | CST-14074S |
| P-YAP | 1:1000 | CST-4911S |
| GAPDH | 1:10000 | ab181602 |
| PCNA | 1:1000 | CST-13110S |
| Col I | 1:1000 | bsm-10423R |
| Col3 | 1:10000 | ab7778 |
| Fn | 1:10000 | ab45688 |
| Smad1/5/9 | 1:1000 | CST-12656T |
| Smad2 | 1:1000 | CST-5339S |
| Smad3 | 1:1000 | CST-9523S |
| Smad2/3 | 1:1000 | CST-8685S |
| ERK1/2 | 1:1000 | bsm-52259R |
| p-ERK1/2 | 1:1000 | CST-4376S |
| MEK1/2 | 1:1000 | CST-4694S |
| p-MEK1/2 | 1:1000 | bsm-52176R |
| LATS1 | 1:1000 | CST-3477S |
| P-LATS1 | 1:1000 | CST-8654S |
| MST1 | 1:1000 | CST-3682S |
| p-MST1 | 1:1000 | bsm-4635R |
